# Fermented *Codonopsis pilosula* residue improved milk performance of lactating donkeys by enhancing antioxidant capacity and regulating metabolism

**DOI:** 10.3389/fvets.2024.1489480

**Published:** 2024-11-18

**Authors:** Miaomiao Zhou, Fei Huang, Xinyi Du, Guiqin Liu, Changfa Wang

**Affiliations:** College of Agriculture and Biology, Liaocheng Research Institute of Donkey High-Efficiency Breeding and Ecological Feeding, Liaocheng University, Liaocheng, China

**Keywords:** donkeys, fermented *Codonopsis pilosula* residue, milk performance, metabolites, blood biochemical parameters

## Abstract

The fermented *Codonopsis pilosula* residue (FCPR) has a promoting effect on animal health, but it has not been studied in livestock feed. This study investigated the effects of FCPR addition on the milk performance, milk metabolites, and blood biochemical parameters and metabolites of lactating donkeys. Twelve healthy multiparous lactating Dezhou donkeys were randomly divided into two groups (with 6 donkeys in each group): control group (without FCPR, C) and FCPR group (200 g of FCPR per head per day, FF). The experiment lasted for 5 weeks. The milk and blood samples were collected at the end of the experiment. The results showed that addition of FCPR significantly increased the daily milk yield and the milk components (protein, lactose, solids, solids-not-fat, and lactoferrin) yield of lactating donkeys and the weight gain of foals (*p* < 0.05). A total of 568 metabolites were detected in donkey milk, including 21 differential metabolites between group FF and group C. Compared to group C, the significantly up-regulated metabolic pathway in the FF group was renin secretion (*p* < 0.05). In addition, the FCPR significantly increased the concentrations of urea, complement C4 (C4), total antioxidant capacity (T-AOC), and catalase (CAT) in the blood, while reducing the concentration of malondialdehyde (MDA) (*p* < 0.05). A total of 753 metabolites were detected in serum of donkeys, including 86 differential metabolites between group FF and group C. Compared to group C, the significantly up-regulated metabolic pathways in the FF group were renin secretion, cAMP signaling pathway, regulation of lipolysis in adipocytes, and fatty acid biosynthesis (*p* < 0.05). The above results indicated that FCPR addition enhance the milk performance of lactating donkeys by activating the immune system, increasing the antioxidant capacity, and improving the glucose and lipid metabolism. These results provide a foundation for the development and utilization of FCPR additives, which is beneficial for livestock production and improving animal welfare.

## 1 Introduction

Chinese herbal medicines (CHM) and plant extractive have been used in livestock and aquaculture and play an important role in animals’ healthy growth (1, 2). Some CHM or Chinese herbal residues (CHR) are added as feed additives to animal diets. It has been found that addition of CHR improved the gut environment of weaned piglets (2). Samanta et al. (3) studied the effects of ginger residue and inulin on pig fecal microbiota and found that they can improve intestinal microbiota and promote healthy growth of pigs. The results of Du et al. (1) found that dietary supplementation of *Taraxacum mongolicum* flavonoids protected *Channa argus* from lipopolysaccharide-induced inflammatory injury, improved digestion, activated immune responses, and enhanced antioxidant capacity. In study of Samanta et al. (3), the effects of *Lonicera japonica* extract on heat-stressed mid-lactation dairy cows were investigated, and the results found that *Lonicera japonica* extract helped to alleviate heat stress by improving antioxidant status and promoting endocrine and immune functions. The results of Abulaiti et al. (4) also showed that CHM supplementation improved the milk yield, milk composition, and serum metabolites in dairy cows.

In addition, fermented Chinese herbal medicines (FCHM) or herbal residues (FCHR) are also widely used in animal husbandry. *Lactic acid bacteria* fermented herbs can regulate immune responses by activating innate or adaptive immune systems and maintaining intestinal immune homeostasis (5). The results of Fujaya et al. (6) showed that the fermented herbal extract enhanced weight gain, growth and feed efficiency of *Nile Tilapia* by increasing nutrient absorption and improving intestinal immune function. FCHM (3 kg/t) improved the weaned piglets growth performance by improving intestinal health and promoting nutrients digestibility (7). The probiotic-fermented herbal blend prevented death and improved growth performance of *Salmonella pullorum* infected chicks by increasing cecal *lactobacilli*, reducing cecal *Escherichia coli* and *Salmonella*, and enhancing immune function (8). The results of Su et al. (2) also found that addition of FCHR improved the gut environment of weaned piglets. In addition, fermented herbal tea residue (FHTR) increased the concentrations of hydroxyl radicals, urea, and glutathione peroxidase (GSH-Px) in serum of goats (9). Zhuang et al. (10) found that FHTR replacing 30% of the forage significantly increased the immunoglobulin G (IgG), total antioxidant capacity (T-AOC), and superoxide dismutase (SOD) levels, changed the fecal microbiota composition, increased the daily feed intake and daily gain of fattening cattle. It was also found that adding FHTR increased the concentration of immunoglobulin A (IgA) and immunoglobulin G (IgG) in serum, as well as the activity of SOD and GSH-Px, thereby improving the growth of heifers (11).

*Codonopsis pilosula*, the root of *C. pilosula (Franch.) Nannf.*, which is known as “Dangshen” in Chinese (12) has been used in food (tea, wine, soup and porridge) and prescribed in traditional folk medicine in Asian countries like China for thousands of years (13, 14). *Codonopsis pilosula* has anti-inflammatory, antioxidant, prebiotic, and immune regulation biological activities, playing an important role in the treatment of cardiovascular diseases, digestive diseases, and immune diseases (15). The *Codonopsis pilosula* polysaccharides (CPPs) are the major bioactive substance with biological activities (13). It was found that CPPs significantly enriched the intestinal probiotic *Lactobacillus* and decreased the abundance of *Enterococcus* and *Shigella*, which was beneficial for the invigorating (16). Research has also found that a polysaccharide extracted from *Codonopsis pilosula* residue also has a regulatory effect on glucose metabolism (17). Furthermore, the *Codonopsis pilosula* extract, inulin-type fructan, reduced the mucosa ulcer index of rats by reducing the malondialdehyde (MDA) and nitric oxide (NO) content and promoting the myeloperoxidase (MPO), superoxide dismutase (SOD), and glutathione peroxidase (GSH-Px) activity (18). It was also found that the *A. membranaceus* and *Codonopsis pilosula* extract mixture improved the growth performance and immunity of growing-finishing pigs (19, 20).

It is speculated that fermented *Codonopsis pilosula* residue (FCPR) also has a promoting effect on animal health. Thus, effects of FCPR on milk performance, milk metabolites, and blood biochemical parameters and metabolites of lactating Dezhou donkeys was investigated in this study.

## 2 Materials and methods

### 2.1 Samples collection and preparation

Milk and serum samples were from 12 healthy multiparous lactating Dezhou donkeys with an age of 3–5 years and an average bodyweight 278 ± 30 kg at 70 ± 5 days in milk (DIM). The donkeys were housed in a farm in Liaocheng City, China. The donkeys were raised in a semi-closed house. Each donkey was kept in a separate pen. All donkeys drank freely and offered the same diet of grass hay (*ad libitum*) supplemented with 2.5 kg concentrate per head per day (Table 1) (21). The donkeys were randomly divided into two groups, with 6 donkeys in each group. One group did not add FCPR (control group, C), while the other group added 200 g of FCPR per head per day (the FCPR group, FF). The fermentation substrate was the *Codonopsis pilosula* residue. And fermentation was conducted using *Bacillus* and *Lactobacillus plantarum* (0.2%). The nutritional composition of the FCPR was as follows: dry matter (50%), crude protein (8%), nitrogen free extract (18%), crude fiber (15%), and ash (9%). The experiment lasted for 5 weeks. The milk and blood samples were collected at the end of the experiment.

**Table 1:** Composition and nutritional level of experimental concentrate (dry-matter basis).

Items	Feed Ingredients (%)
Corn	31.75
Wheat bran	12.00
Wheat flour middling	21.00
DDGS	5.00
Wheat germ	18.00
Soybean meal	6.65
NaCl	0.63
CaCO_3_	3.72
CaHPO_4_	0.25
Premix^1^	1.00
Total	100
	Nutrient level (%)
Dry matter	88.53
Crude protein	17.18
Ash	8.37
Crude fiber	4.27
Ether extract	4.59
Calcium	1.27
Phosphorus	0.52
Lysine	0.75

1 Premix/kg: VA, 600 KIU; VD3, 125 KIU; VE, 3500 IU; Fe, 2 g; Cu, 800 mg; Zn, 6 g; Mn, 13 g; I, 95 mg; Se, 50 mg.

The lactating donkeys were manual-milked twice a day (11 am and 3 pm). The foals were removed from the mother for 4 h before the milking. The milk yield of the donkey was recorded. The milk samples were collected during milking twice a day, frozen quickly in liquid nitrogen and stored at −80°C until analysis. The blood samples were collected from the jugular vein before feeding in the morning. After stratification, the blood samples were centrifuged at 3500 rpm, 4°C for 15 min to separate the serum. Then, the serum was frozen quickly with liquid nitrogen and stored at −80°C.

### 2.2 Milk composition determination

The milk compositions (fat, protein, lactose, urea, lactoferrin, solids, and solids-not-fat) were determined using the CombiScope FTIR300 (Delta Instruments B.V., Drachten, The Netherlands). There were three replicates for each sample.

### 2.3 Measurement of blood biochemical parameters

The concentration or international units (IU) of serum glucose (GLU), total protein (TP), albumin (ALB), triglyceride (TG), urea, aspartate aminotransferase (AST), alanine aminotransferase (ALT), lactate dehydrogenase (LDH), alkaline phosphatase (ALP), complement C3 (C3), complement C4 (C4), IgA, IgG, immunoglobulin M (IgM), SOD, GSH-Px, T-AOC, catalase (CAT), and MDA were measured using the automatic biochemical analyzer (Mindray BS-420, Shenzhen, China), and the test kits were provided by BioSino Bio-Technology and Science Inc. (Beijing, China).

### 2.4 Detection of milk and serum metabolites

#### 2.4.1 Metabolites extraction

Milk or serum sample (100 μL) was added with prechilled 80% methanol. Then, the sample was placed into the Eppendorf tube and vortexed. Afterwards, the samples were incubated on ice for 5 min. Then the samples were centrifuged for 20 min at 15,000 g, 4°C. Some of supernatant was diluted with LC–MS grade water to the final concentration containing 53% methanol. After that, the samples were transferred to a fresh Eppendorf tube and then were centrifuged for 20 min at 15,000 g, 4°C. Finally, the supernatant was analyzed by liquid chromatography–tandem mass spectrometry (LC–MS/MS).

#### 2.4.2 UHPLC–MS/MS analysis

The Vanquish UHPLC system (Thermo Fisher, Germany) coupled with an Orbitrap Q Exactive TM HF mass spectrometer (Thermo Fisher, Germany) were used in UHPLC–MS/MS analyses. Samples were injected onto a Hypersil Goldcolumn (100 × 2.1 mm, 1.9 μm) at a flow rate of 0.2 mL/min using a 12-min linear gradient. The eluents for the positive and negative polarity modes were eluent A (0.1% formic acid in Water) and eluent B (methanol). The solvent gradient was set as follows: 2% B, 1.5 min; 2–85% B, 3 min; 85–100% B, 10 min; 100–2% B, 10.1 min; 2% B, 12 min. Q Exactive TM HF mass spectrometer was operated in positive/negative polarity mode with spray voltage of 3.5 kV, capillary temperature of 320°C, sheath gas flow rate of 35 psi and aux gas flow rate of 10 L/min, S-lens RF level of 60, Aux gas heater temperature of 350°C.

#### 2.4.3 Data processing and metabolite identification

The Compound Discoverer 3.3 (CD3.3, ThermoFisher) was used to process the raw data files for peak alignment, peak picking, and quantitation for each metabolite. After that, peak intensities were normalized to the total spectral intensity. Then predicted the molecular formula using the normalized data based on additive ions, molecular ion peaks and fragment ions. And then peaks were matched with the mzCloud,^1^ mzVault and MassList database to obtain the accurate qualitative and relative quantitative results. The statistical software R (R version R-3.4.3), Python (Python 2.7.6 version) and CentOS (CentOS release 6.6) were used in statistical analyses. Throughout the analytical run, aliquots of all the milk or plasma samples were mixed to create quality control (QC) samples, which were injected at regular intervals to assess the stability of the analytical system.

### 2.5 Data analysis

These metabolites were annotated using the Kyoto Encyclopedia of Genes and Genomes (KEGG) database,^2^ HMDB database^3^ and LIPIDMaps database.^4^ Principal components analysis (PCA) and Partial least squares discriminant analysis (PLS-DA) was performed at metaX. The statistical significance (*p*-value) was calculated using univariate analysis (*t*-test). The metabolites were considered to be differential metabolites when VIP > 1, *p* < 0.05, and fold change (FC) ≥ 2 or FC ≤ 0.5. For clustering heat maps, the data were normalized using z-scores of the intensity areas of differential metabolites and were ploted by Pheatmap package in R language. The functions of these metabolites and metabolic pathways were studied using the KEGG database. The metabolic pathways enrichment of differential metabolites was performed, when ratio was satisfied by x/n > y/N and *p* < 0.05, the metabolic pathway was considered as statistically significant enrichment. The Pearson correlation coefficient R^2^ between QC samples was between 0.991 and 0.996, which was very close to 1, indicating good stability and high data quality of the detection process.

The other data were analyzed by *t*-test in GraphPad Prism 6 software. The results were shown as mean ± SD. The significant difference was defined when *p* < 0.05.

## 3 Results

### 3.1 Effects of FCPR on milk performance of lactating donkeys

The FCPR had no effect on the average daily feed intake of lactating donkeys, and had significant impacts on donkey milk yield, foals’ weight gain, and milk component yield (Table 2). The donkey daily milk yield and foals’ weight gain in FF group were significantly higher than that in the C group (*p* < 0.05). Compared with the control group, the weight gain of lactating donkeys in the FF group showed an increasing trend (*p* = 0.0909). As for milk composition, there was no difference between the two groups. However, except for milk fat and polyunsaturated fatty acid, the yield of other milk components such as protein, lactose, solids, solids-not-fat, and lactoferrin in the FF group was significantly higher than that in the control group (*p* < 0.05).

**Table 2:** Effects of fermented herbal residue on milk performance of lactating donkeys.

Items	C	FF	P
Average daily feed intake (kg)	5.54 ± 0.45	5.73 ± 0.44	0.5598
Daily milk yield (mL)	1,103 ± 187^b^	1,452 ± 280^a^	0.0295
Weight gain of foals (kg)	11.75 ± 4.68^b^	19.83 ± 7.50^a^	0.0490
Weight gain of lactating donkeys (kg)	−0.67 ± 9.68	13.33 ± 15.57	0.0909
Milk composition			
Milk fat (%)	0.34 ± 0.42	0.32 ± 0.31	0.9208
Milk protein (%)	1.57 ± 0.18	1.54 ± 0.12	0.6946
Milk lactose (%)	6.53 ± 0.29	6.64 ± 0.08	0.4132
Solids (%)	9.18 ± 0.34	9.23 ± 0.32	0.8009
Solids-not-fat (%)	8.87 ± 0.17	8.96 ± 0.16	0.3636
Urea nitrogen (mg/dL)	13.51 ± 3.31	15.23 ± 2.41	0.3300
Polyunsaturated fatty acid (%)	0.21 ± 0.02	0.20 ± 0.01	0.7976
Lactoferrin (g/L)	0.095 ± 0.0031	0.096 ± 0.0032	0.5593
Daily milk component yield			
Milk fat (g)	3.73 ± 4.61	5.20 ± 5.52	0.6253
Milk protein (g)	17.26 ± 2.93^b^	22.32 ± 4.73^a^	0.0499
Milk lactose (g)	72.01 ± 13.04^b^	96.18 ± 17.62^a^	0.0223
Solids (g)	101.13 ± 16.93^b^	134.44 ± 29.01^a^	0.0355
Solids-not-fat (g)	97.62 ± 15.87^b^	129.84 ± 24.05^a^	0.0208
Polyunsaturated fatty acid (g)	2.28 ± 0.43	2.98 ± 0.67	0.0578
Lactoferrin (mg)	104.69 ± 16.93^b^	140.03 ± 29.86^a^	0.0302

a,b Means within a row with different superscripts were significant (*p* < 0.05).

### 3.2 Effects of FCPR on milk metabolites

**Figure 1:**
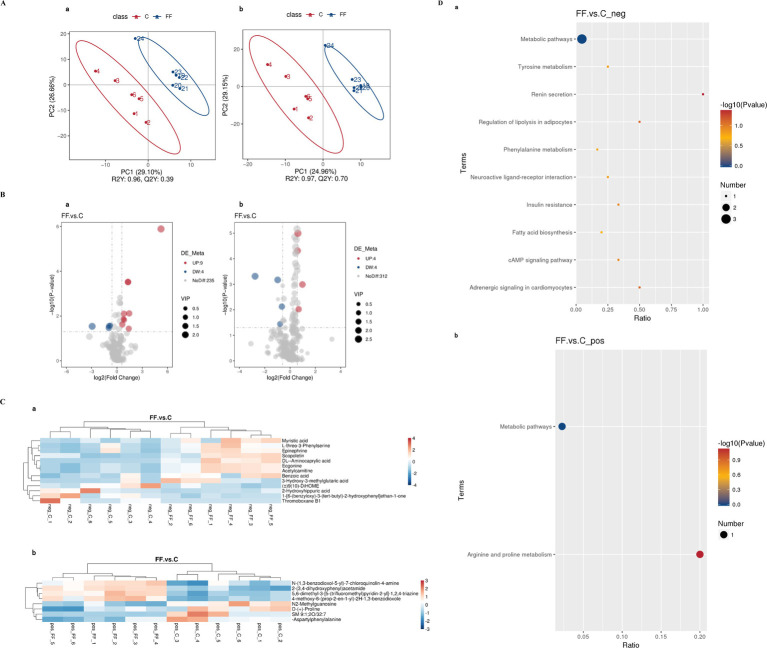
The PLS-DA analysis **(A)** of milk metabolites and volcano plots **(B)**, heatmap **(C)**, and the involved metabolic pathways **(D)** of donkey milk differential metabolites between group FF and group C. a (b): the negative (positive) ion mode. **(A)** The horizontal axis PC1 and vertical axis PC2 represent the scores of the principal components ranked first and second, respectively. Different colored dots represent samples from different groups. Ellipses represent a 95% confidence interval. **(B)** FC = fold change. **(C)** Red indicates functions with relatively high values (0–4/3), while blue represents a relatively low value [0-(−4)/(−3)]. The milk samples were taken from 12 lactating donkeys with (FF) or without fermented *Codonopsis pilosula* residue addition (C).

The data of milk metabolites from donkeys with or without FCPR were analyzed using PLS-DA. Figure 1A displayed the PLS-DA score plot of milk metabolites from groups C and FF. Figures 1B,C showed the volcano plots and heatmap of differential metabolites in different groups of donkey milk. A total of 568 metabolites were detected in donkey milk, including 21 differential metabolites, of which 13 were up-regulated and 8 were down-regulated. The differential metabolites were displayed in Table 3 (*p* < 0.05). The main metabolic pathways associated with the differential metabolites and the metabolic pathways classification were shown as Figure 1D. Compared to group C, the significantly up-regulated metabolic pathway in the FF group was renin secretion (*p* < 0.05). And the metabolic pathways of arginine and proline metabolism (*p* = 0.0735), adrenergic signaling in cardiomyocytes (*p* = 0.0804), and regulation of lipolysis in adipocytes (*p* = 0.0804) showed an increasing trend.

**Table 3:** Differential metabolites of donkey milk between group FF and group C.

Regulated type	Name	Formula	Molecular weight	RT^a^ (min)	FC^b^
Up-regulated	DL-α-Aminocaprylic acid	C8 H17 NO2	159.1264	5.604	37.4205
	Scopoletin	C10 H8 O4	192.0427	5.25	2.74600
	3-Hydroxy-3-methylglutaric acid	C6 H10 O5	162.0531	1.397	2.6382
	Ecgonine	C9 H15 N O3	185.1056	5.754	2.4805
	Acetylcarnitine	C9 H17 N O4	203.1161	5.756	2.4805
	2-(3,4-dihydroxyphenyl)acetamide	C8 H9 N O3	167.0593	1.962	1.9689
	Myristic acid	C14 H28 O2	228.2098	0.228	1.8147
	L-threo-3-Phenylserine	C9 H11 N O3	181.0743	5.519	1.7938
	Epinephrine	C9 H13 N O3	183.0899	5.663	1.7033
	N-(1,3-benzodioxol-5-yl)-7-chloroquinolin-4-amine	C16 H11 Cl N2 O2	298.047	1.284	1.6033
	Benzoic acid	C7 H6 O2	122.0372	5.95	1.5601
	5,6-dimethyl-3-[5-(trifluoromethyl)pyridin-2-yl]-1,2,4-triazine	C11 H9 F3 N4	254.0793	3.137	1.5389
	4-methoxy-6-(prop-2-en-1-yl)-2H-1,3-benzodioxole	C11 H12 O3	192.0805	8.257	1.5104
Down-regulated	Thromoboxane B1	C20 H36 O6	408.2313	9.406	0.1309
	SM 9:1;2O/32:7	C46 H79 N2 O6 P	786.5745	10.126	0.1472
	N2-Methylguanosine	C11 H15 N5 O5	297.1088	4.808	0.5076
	2-Hydroxyhippuric acid	C9 H9 N O4	195.0536	5.008	0.5161
	(±)9(10)-DiHOME	C18 H34 O4	314.2462	10.745	0.5236
	D-(+)-Proline	C5 H9 N O2	115.0641	1.446	0.5793
	1-[6-(benzyloxy)-3-(tert-butyl)-2-hydroxyphenyl]ethan-1-one	C19 H22 O3	298.1611	8.605	0.5780
	α-Aspartylphenylalanine	C13 H16 N2 O5	280.1065	5.348	0.6387

a RT, retention time.

b FC, fold change, mean value of peak area obtained from group FF/mean value of peak area obtained from group C.

### 3.3 Effects of FCPR on blood biochemical parameters

The changes in blood biochemical parameters of lactating donkeys were shown in Table 4. The addition of FCPR to the diet of lactating donkeys significantly increased the blood urea and C4 concentration and activity of T-AOC, CAT (*p* < 0.05). And the concentration of MDA of group FF was significantly lower than group C (*p* < 0.01). Compared with the control group, the activity of SOD in the FF group showed an increasing trend (*p* = 0.0885). The other detected blood biochemical parameters were not affected by FCPR addition.

**Table 4:** Blood biochemical parameters analysis between group FF and group C.

Items	C	FF	P
TP (g/L)	62.91 ± 5.94	58.71 ± 3.48	0.1660
ALB (g/L)	23.43 ± 1.76	23.33 ± 1.40	0.9166
TG (mmol/L)	0.17 ± 0.07	0.14 ± 0.05	0.4261
Urea (mmol/L)	7.69 ± 0.32^b^	8.53 ± 0.75^a^	0.0299
GLU (mmol/L)	4.29 ± 0.60	4.37 ± 0.39	0.7783
AST (U/L)	270.55 ± 21.05	298.35 ± 42.88	0.1844
ALT (U/L)	6.41 ± 3.16	9.81 ± 8.03	0.3568
LDH (U/L)	293.02 ± 71.06	284.55 ± 45.94	0.8114
ALP (U/L)	18.64 ± 21.69	13.229 ± 8.71	0.5825
C3 (g/L)	1.34 ± 0.51	1.49 ± 0.65	0.6557
C4 (g/L)	0.39 ± 0.06^b^	0.71 ± 0.19^a^	0.0030
IgA (g/L)	2.03 ± 0.38	2.10 ± 0.19	0.7233
IgG (g/L)	19.19 ± 2.46	19.87 ± 1.95	0.6035
IgM (g/L)	2.90 ± 0.55	2.93 ± 0.49	0.9434
SOD (U/mL)	72.64 ± 12.00	85.45 ± 11.52	0.0885
GSH-Px (U/mL)	182.95 ± 27.00	207.06 ± 45.60	0.2910
T-AOC (U/mL)	8.83 ± 0.33^b^	10.04 ± 0.34^a^	< 0.0001
CAT (U/mL)	33.59 ± 7.22^b^	66.56 ± 12.11^a^	0.0002
MDA (nmol/mL)	4.05 ± 0.67^a^	3.16 ± 0.40^b^	0.0191

^a,b^Means within a row with different superscripts were significant (*P* < 0.05).

### 3.4 Effects of FCPR on blood metabolites

**Figure 2:**
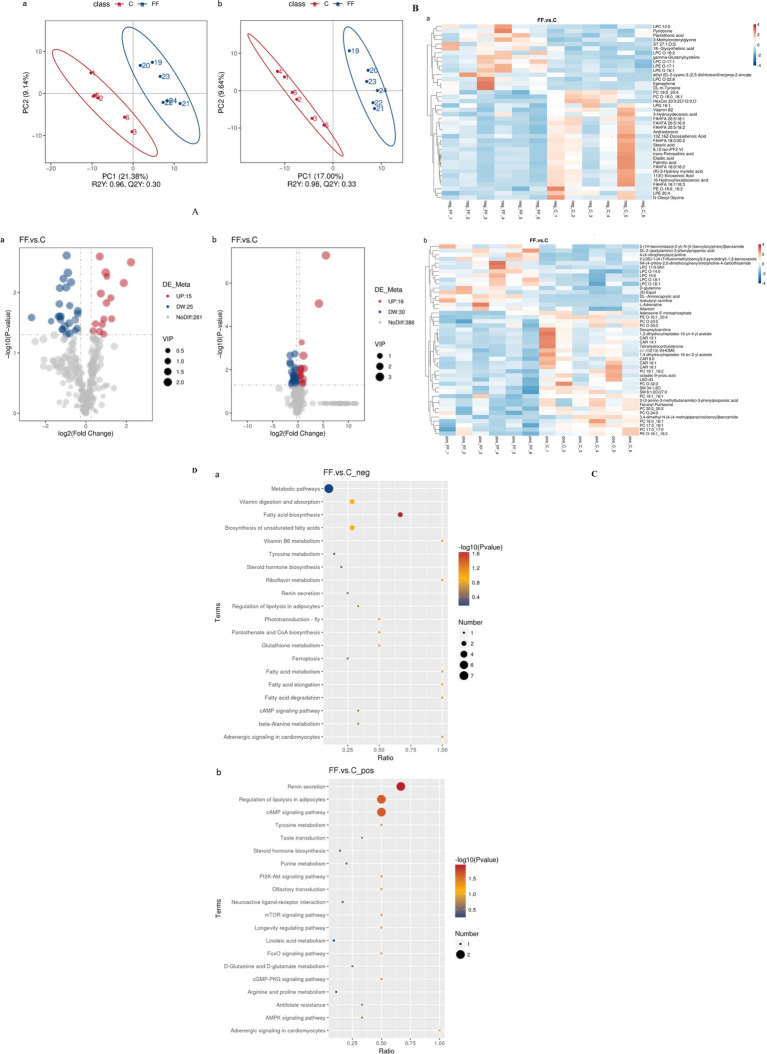
The PLS-DA analysis **(A)** of serum metabolites and volcano plots **(B)**, heatmap **(C)**, and the involved metabolic pathways **(D)** of donkey serum differential metabolites between group FF and group C. a (b): the negative (positive) ion mode. **(A)** The horizontal axis PC1 and vertical axis PC2 represent the scores of the principal components ranked first and second, respectively. Different colored dots represent samples from different groups. Ellipses represent a 95% confidence interval. **(B)** FC = fold change. **(C)** Red indicates functions with relatively high values (0–4), while blue represents a relatively low value [0-(−4)]. The serum samples were taken from 12 lactating donkeys with (FF) or without fermented *Codonopsis pilosula* residue addition (C).

The data of serum metabolites from donkeys with or without FCPR were analyzed using PLS-DA. Figure 2A displayed the PLS-DA score plot of serum metabolites from groups C and FF. Figures 2B,C showed the volcano plots and heatmap of differential metabolites in different groups of donkey serum. A total of 753 metabolites were detected in donkey serum, including 86 differential metabolites, of which 31 were up-regulated and 55 were down-regulated. Some differential metabolites were shown in Table 5 (FC > 2, *p* < 0.05). The main metabolic pathways associated with the differential metabolites and the metabolic pathways classification were shown as Figure 2D. Compared to group C, the significantly up-regulated metabolic pathways in the FF group included renin secretion, cAMP signaling pathway, regulation of lipolysis in adipocytes, and fatty acid biosynthesis (*p* < 0.05). And the metabolic pathways of fatty acid elongation, fatty acid degradation, riboflavin metabolism, vitamin B6 metabolism, fatty acid metabolism, and adrenergic signaling in cardiomyocytes showed an increasing trend (*p* = 0.0885).

**Table 5:** Differential metabolites of donkey serum between group FF and group C.

Regulated type	Name	Formula	Molecular weight	RT^a^ (min)	FC^b^
Up-regulated	DL-α-Aminocaprylic acid	C8 H17 N O2	159.1264	5.621	47.0596
	Isobutyryl carnitine	C11 H21 N O4	231.1477	5.443	17.8705
	Epinephrine	C9 H13 N O3	183.0897	5.662	4.6961
	DL-m-Tyrosine	C9 H11 N O3	181.0738	5.5	3.6673
	Ethyl (E)-2-cyano-3-(2,5-dichloroanilino)prop-2-enoate	C12 H10 Cl2 N2 O2	284.0142	11.158	2.4192
	L-Adrenaline	C9 H13 N O3	183.0901	5.673	2.3813
	LPC O-22:6	C30 H52 N O6 P	599.3502	7.279	2.3527
	4-(4-nitrophenylazo)aniline	C12 H10 N4 O2	242.0774	1.228	2.1387
	2-{(3S)-1-[4-(Trifluoromethyl)benzyl]-3-pyrrolidinyl}-1,3-benzoxazole	C19 H17 F3 N2 O	346.125	1.26	2.0916
	Gamma-Glutamylcysteine	C8 H14 N2 O5 S	250.0631	6.24	2.0527
	LPC 12:0	C20 H42 N O7 P	485.2761	7.845	2.0275
Down-regulated	PC O-33:2	C41 H80 N O7 P	747.5795	10.437	0.6613
	PC O-30:0	C38 H78 N O7 P	691.5531	10.59	0.6588
	PC 16:0_16:1	C40 H78 N O8 P	731.5447	8.884	0.6457
	SM 34:1;2O	C39 H79 N2 O6 P	724.5518	11.165	0.6119
	(+/−)12(13)-DiHOME	C18 H34 O4	296.2358	8.235	0.6057
	Stearic acid	C18 H36 O2	284.2716	11.158	0.6021
	CAR 8:0	C15 H30 N O4	287.2102	6.156	0.5995
	8,12-iso-iPF2α-VI	C20 H34 O5	336.228	10.129	0.5751
	LPG 16:1	C22 H43 O9 P	482.2649	8.81	0.5687
	Palmitic acid	C16 H32 O2	256.2403	10.337	0.5646
	PC 20:2_20:2	C48 H88 N O8 P	837.6267	11.465	0.5503
	Tetrahydrocorticosterone	C21 H34 O4	385.2837	7.16	0.5450
	Vitamin B2	C17 H20 N4 O6	376.1397	9.525	0.5406
	1,4-dihydroxyheptadec-16-en-2-yl acetate	C19 H36 O4	328.2615	8.211	0.5333
	Decanoylcarnitine	C17 H33 N O4	315.2414	6.688	0.5294
	FAHFA 18:0/20:2	C38 H70 O4	590.5259	11.158	0.5177
	FAHFA 16:0/18:2	C34 H62 O4	534.4628	10.337	0.5154
	11(E)-Eicosenoic Acid	C20 H38 O2	310.2875	11.333	0.5086
	PC O-34:0	C42 H86 N O7 P	747.6094	10.301	0.4911
	PE O-18:0_18:2	C41 H80 N O7 P	729.5662	8.866	0.4874
	CAR 18:1	C25 H48 N O4	425.3514	8.546	0.4704
	(R)-3-Hydroxy myristic acid	C14 H28 O3	244.2039	7.45	0.4642
	FAHFA 20:5/18:2	C38 H60 O4	580.45	9.505	0.4472
	Androsterone	C19 H30 O2	290.2249	9.503	0.4472
	1,2-dihydroxyheptadec-16-yn-4-yl acetate	C19 H34 O4	343.273	7.24	0.4283
	Trans-Petroselinic acid	C18 H34 O2	328.2616	10.525	0.4254
	Elaidic acid	C18 H34 O2	282.2558	10.528	0.4192
	Adenosine 5′-monophosphate	C10 H14 N5 O7 P	347.0635	1.397	0.4103
	N-Oleoyl Glycine	C20 H37 N O3	339.2777	9.651	0.4080
	PC O-16:0_18:1	C42 H84 N O7 P	791.6055	9.904	0.4079
	16-Hydroxyhexadecanoic acid	C16 H32 O3	254.2247	9.724	0.4030
	FAHFA 20:5/16:0	C36 H60 O4	556.4495	9.741	0.3890
	CAR 16:1	C23 H44 N O4	397.3199	7.974	0.3588
	CAR 12:1	C19 H36 N O4	341.257	6.931	0.3470
	CAR 14:1	C21 H40 N O4	387.2991	7.471	0.3405
	HexCer 20:0;2O/12:0;O	C38 H75 N O9	749.5789	8.619	0.3035
	FAHFA 16:1/18:3	C34 H58 O4	530.4317	9.722	0.1550

a RT, retention time.

b FC, fold change, mean value of peak area obtained from group FF/mean value of peak area obtained from group C.

## 4 Discussion

Some studies have shown that adding FCHM to the diet can improve animal production performance. It was found that addition of FCHM improved the growth performance of weaned piglets (7). Addition of FHTR improved the rumen fermentation, and thus affected serum indicators and meat quality of black goats (22). The results of Zhuang et al. (10) showed that FHTR significantly increased the daily feed intake and daily gain of fattening cattle. In study of Shan et al. (23), the effects of dietary FCHM supplementation on milk performance were investigated, and the results found that FCHM improved the milk yield, milk fat and protein content, and immune function of dairy cows under heat stress conditions. In this study, the addition of FCPR significantly increased the donkeys’ milk yield, weight gain of foals, and the milk components (protein, lactose, solids, solids-not-fat, and lactoferrin) yield (*p* < 0.05). And FCPR also changed the metabolites in donkey milk. A total of 21 differential metabolites in donkey milk were detected between FF and C groups. Compared to group C, the significantly up-regulated metabolic pathway in the FF group was renin secretion. The composition of donkey milk is influenced by various factors such as breed, lactation stage, season, and dietary composition (24–26). In this study, the milk was from 12 healthy multiparous lactating Dezhou donkeys with the similar age, weight, and lactation stage. In addition, except for FCPR, lactating donkeys were fed with the same diet. Thus, the above results indicated that FCPR improved the lactation performance of female donkeys and altered the metabolites of donkey milk. Our results were consistent with the previous conclusion.

It has been found that *Codonopsis pilosula* has anti-inflammatory, antioxidant, and immune regulation biological activities (13, 15). Sun and Liu (27) investigated the immunity activity of CPPs, and the results found that it can stimulated lymphocyte proliferation in a dose-dependent manner. The CPPs exerted antitumor activity by enhancing immune system function (28). Results of Fan et al. (29) showed that *Codonopsis pilosula* glucofructan (CPG) improved both the humoral and cellular immunity and effectively inhibited tumor growth in mice. CPG could enhance immunoregulatory function and can be used as a functional food for humans (30). In this study, FCPR exhibited similar immunomodulatory biological activity. Addition of FCPR improved the immune function of lactating donkeys by increasing the level of C4 in the blood, thereby enhancing their milk production performance.

Furthermore, many studies have also shown *Codonopsis pilosula* has antioxidant activity. *Codonopsis pilosula* extract significantly alleviated mitochondrial damage and activated autophagy, thereby improving hippocampal tissue damage and alleviating cognitive impairment in aging mice (31). CPPs significantly reduced serum blood glucose, insulin level and MDA content increased SOD activity, and significantly improved insulin resistance of diabetes mice (32). In study of Liu et al. (33), the polysaccharides increased the activities of total SOD and GSH-Px, and decreased the level of MDA, and thereby increased the BW of red swamp crayfish. The neutral polysaccharide (CPP-1) could protect cells from oxidative damage (increasing the activity of SOD and CAT and reducing the MDA content), reduce the body fat index of nonalcoholic fatty liver disease mice, and improve the liver function (34). Results of other studies also found CPPs exerts a protective effect by enhancing antioxidant activity (increasing GSH-Px, SOD, and T-AOC activity and reducing MDA levels), regulating the dynamic balance of NO and endothelin-1, and significantly improving the survival rate of cells treated with hydrogen peroxide (35–37). The SOD, GSH Px, and CAT are antioxidant enzymes that catalyze the conversion of free superoxide anion radicals into non-toxic compounds, which are crucial for reducing oxidative stress and maintaining animal health (38). In this study, the addition of FCPR to the diet of lactating donkeys significantly increased the concentrations of T-AOC and CAT in the blood, while reducing the concentration of MDA (*p* < 0.01). Our findings were consistent with the conclusions of other studies. Our results indicated that FCPR improved the milk performance of lactating donkeys by enhancing their blood antioxidant capacity.

In addition, the blood metabolites of lactating donkeys were also detected in this study. A total of 753 metabolites were detected in donkey serum, including 86 differential metabolites, of which 31 were up-regulated and 55 were down-regulated. The main up-regulated differential metabolites including DL-*α*-aminocaprylic acid, isobutyryl carnitine, epinephrine, DL-m-tyrosine, ethyl (E)-2-cyano-3-(2,5-dichloroanilino)prop-2-enoate, L-Adrenaline, LPC O-22:6, 4-(4-nitrophenylazo) aniline, 2-{(3S)-1-[4-(trifluoromethyl) benzyl]-3-pyrrolidinyl}-1,3-benzoxazole, gamma-glutamylcysteine, and LPC 12:0 (FC > 2, *p* < 0.05). The differential metabolic pathways associated with these differential metabolites were renin secretion, cAMP signaling pathway, regulation of lipolysis in adipocytes, and fatty acid biosynthesis (p < 0.05). The above metabolic pathways were mainly related to glucose metabolism and lipid metabolism. It has been found that CPPs improved the lipid metabolism and glucose metabolism impairment, and reduce insulin resistance of obesity mice, and has the potential for the treatment of obesity (39). Bai et al. (40) investigated the effects of *Codonopsis pilosula* oligosaccharides (CPO) supplementation on obesity, and the results found that CPO decreased body weight and fat accumulation and improved glucose tolerance in high-fat diet induced obese mice. The results of Hu et al. (41) also found that *Codonopsis pilosula* polyynes participated in lipid metabolism by inhibiting the expression of squalene monooxygenase gene, which may be used in treatment of hypercholesterolemia and atherosclerosis. Liu et al. (17) also found that the polysaccharide extracted from *Codonopsis pilosula* residue improved the glucose metabolism by alleviating oxidative stress, improving lipid metabolism, and increasing glycolytic enzymes activity. Therefore, in this study, FCPR may enhance the milk performance of lactating donkeys by improving glucose and lipid metabolism and enhancing energy utilization efficiency.

In summary, the addition of FCPR significantly increased the daily milk yield, the yield of milk protein, lactose, solids, solids-not-fat, and lactoferrin, as well as the foals’ weight gain. A total of 21 differential metabolites between FF and C groups, which were related to global and overview maps, amino acid metabolism, and endocrine system metabolic pathways. The addition of FCPR also significantly increased the concentrations of urea, C4, T-AOC, and CAT in the blood, while reducing the concentration of MDA. And 86 differential metabolites were detected between FF and C groups, which were associated with renin secretion, cAMP signaling pathway, regulation of lipolysis in adipocytes, and fatty acid biosynthesis. In conclusion, FCPR may enhance the milk performance of lactating donkeys by activating the immune system, increasing the antioxidant capacity, and improving the glucose and lipid metabolism. These results provide a foundation for the development and utilization of FCPR additives, which are beneficial for livestock production and improve animal welfare.

## Data Availability

The original contributions presented in the study are included in the article/supplementary material, further inquiries can be directed to the corresponding authors.

## Footnotes

^1^https://www.mzcloud.org/

^2^https://www.genome.jp/kegg/pathway.html

^3^https://hmdb.ca/metabolites

^4^http://www.lipidmaps.org/
